# O081: Reduction of clostridium difficile infections with enhanced evidence-based practices and antimicrobial stewardship

**DOI:** 10.1186/2047-2994-2-S1-O81

**Published:** 2013-06-20

**Authors:** K Alexander-Mills, A Kish, F Palmieri, A Foster, D Viola, F Petersen-Fitzpatrick

**Affiliations:** 1 Bronx Lebanon Hospital Center, Bronx, USA; 2 New York Medical College, Valhalla, USA

## Introduction

Infection rates, deaths, and excess healthcare costs resulting from *Clostridium difficile* infection (CDI) in hospitalized patients are at an historic high, costing the United States Healthcare system at least an extra $1 billion annually. This study assesses the incidence of CDI cases at an inner city hospital in the Northeast Tristate region and determines whether evidence-based intervention and antimicrobial stewardship showed a reduction in CDI.

## Methods

A retrospective review of cases with CDI in this facility from January 2010 to December 2011 was conducted in relation to evidence-based practices and antimicrobial stewardship intervention. In 2008, an evidence-based bundle was implemented in cooperation with the New York State Department of Health (NYSDOH). The bundle consisted of limited adherence to hand washing, environmental cleaning, and isolating the patient. In 2010 this practice was modified with the incorporation of a fledging antimicrobial stewardship program. The antimicrobial stewardship program is designed to use the hospital antibiogram to empirically treat diseases with the intention of optimizing antimicrobial use according to the susceptibility patterns of the organisms.

## Results

Prior to implementation of the interventions there were 186 cases of CDI in 2010. After introducing the modified bundle, there were 121 cases post intervention in 2011. The empiric treatment of certain medical conditions per patient days of therapy with antimicrobial agents called quinolones was decreased with the addition of the antimicrobial stewardship program. The decrease in days of therapy of quinolones by 75% led to a reduction in CDI cases by 39% and a decrease in the overall length of stay from 6.83 in 2010 to 6.38 in 2011.

## Conclusion

The findings from this study provide further evidence that evidence-based practices when followed appropriately can reduce the acquisition and transmission of CDI. Decreasing CDI requires a multifaceted approach including formulary restrictions, staff education, and guidelines that will improve patient safety, decrease healthcare costs and enhance quality of care.

## Disclosure of interest

None declared.

